# Distinct clinical characteristics draw a new prognostic model for splenic marginal zone lymphoma in HBV high prevalent region

**DOI:** 10.18632/oncotarget.21931

**Published:** 2017-10-19

**Authors:** Shuhua Yi, Yuting Yan, Wenjie Xiong, Rui Lv, Zhen Yu, Wei Liu, Enbin Liu, Heng Li, Huimin Liu, Zengjun Li, Gang An, Yan Xu, Kun Ru, Dehui Zou, Lugui Qiu

**Affiliations:** ^1^ State Key Laboratory of Experimental Hematology, Institute of Hematology & Blood Diseases Hospital, Chinese Academy of Medical Science & Peking Union Medical College, Tianjin 300020, China

**Keywords:** splenic marginal zone lymphoma (SMZL), hepatitis B virus (HBV), cytogenetic aberration, IGH gene, prognosis

## Abstract

Splenic marginal zone lymphoma (SMZL) is a rare indolent B-cell neoplasm with hepatitis virus supposed to involve in the pathogenesis. The characteristics of SMZL derived from Caucasia population and high hepatitis C virus (HCV) infection region have been widely investigated, but few was reported in the Eastern population with HBV prevalent region. We analyzed the clinical characteristics, cytogenetic aberrations and prognostic factors in 160 SMZL patients from China. 25 patients (16%) were HBsAg-positive and 54 (34%) patients with resolved HBV infection. IGH gene usage was analyzed in 39 patients. The preferential usages of IGHV genes were IGHV1-2 (26%), followed by IGHV4-34 (18%) and IGHV2-70 (10%). The patients with HBV infection presented biased IGHV-D-J rearrangements and mutational status. Using three independent factors hemoglobin level, HBsAg positivity and complex karyotype, we developed a new hierarchical prognostic model, which showed a better c-index than the previously reported IIL and HPLL scoring systems in SMZL. In conclusion, SMZL in HBV prevalent region have unique clinical and biological characteristics and new prognostic scoring model should be adopted in this population.

## INTRODUCTION

Splenic marginal zone lymphoma (SMZL) is a rare indolent B-cell lymphoma listed as a distinct clinical and pathologic entity in the World Health Organization (WHO) classification of lymphoid neoplasm [1]. Patients with SMZL often have an indolent course with a median overall survival (OS) in excess of 10 years. However, about a quarter of patients display a more aggressive course and die within 3 years after diagnosis [2]. Given the rarity of this disease, the clinical presentation, etiology, consensus treatment, prognostic system and pathogenesis of SMZL have remained controversial.

Exhilaratingly, attentions have been increasingly paid on this disease in the past 5 to 10 years, in both clinical and biological aspects. For example, deletion of 7q, trisomy 3 and gain of 12q have been identified as the most frequent cytogenetic abnormalities in SMZL [3]. The immunoglobulin heavy variable (IGHV) gene analysis showed that biased usage of IGHV1, IGHV3 and IGHV4 were found, indicating possible chronic antigenic stimulations in the generation of SMZL [4].

However, the conclusions mentioned above all come from the Caucasia population and some with high HCV infection. Previously we reported that Chinese patients with SMZL had a significant association with HBV infection [5]. The relative data of SMZL from the Eastern population is scarce and the situations may be different, especially in HBV epidemic region, because the genetic predisposition has been identified to be important in the tumourgenesis and chronic hepatitis virus stimulation has been thought to play important role in the pathogenesis of SMZL. In this study, we described the clinical characteristics of 160 Chinese SMZL patients, analyzed the usage of IGHV/D/J genes and the cytogenetic aberrations, and finally drew a new prognostic system.

## RESULTS

### Clinical characteristics and immunophenotype

The baseline clinical characteristics were summarized in Table 1. The median age at diagnosis was 57 years (range, 25-80 years). The majority had stage IV disease (157/160, 98%). Extranodal tissue involvement was detected in 29 patients included liver (n = 25), parotid gland (n = 2), and central nervous system (n = 2). Both of the two patients with central nervous system involvement had transformed to diffuse large B cell lymphoma. Autoimmune manifestations were observed in 17 patients (11%) included autoimmune hemolytic anemia (n=11), sjogren syndrome (n=2), rheumatoid arthritis (n=2), vasculitis (n=1), polymyositis (n=1).

**Table 1:** The clinical and cytogenetic characteristics of SMZL and the univariate analysis of the prognostic factors

	Characteristics	n	%	5y-FFS, %(SE)	Log rank p value	5y-OS, %(SE)	Log rank p value
**Clinical Characteristics**	**Sex**	160			0.468		0.483
	Female		49	57.1(7.8)		78.1(5.1)	
	Male		51	58.4(6.2)		84.0(5.1)	
	**Age**	160			0.693		0.116
	< 60 y		61	57.3(6.0)		86.8(3.8)	
	≥ 60 y		39	61.4(7.4)		70.7(7.6)	
	**B symptoms**	160			0.857		0.480
	Absence		57	60.1(6.2)		81.7(5.0)	
	Presence		43	55.0(7.7)		80.1(5.3)	
	**Liver involvement**	160			0.778		0.291
	Absence		84	60.0(5.1)		81.4(4.0)	
	Presence		16	49.1(12.6)		79.1(8.3)	
	**ECOG performance status**	160			**0.004**		**0.001**
	0-1		81	76.5(4.0)		89.9(2.9)	
	≥ 2		19	35.0(10.3)		61.1(9.3)	
	**Hemoglobin level**	160			**0.000**		**0.000**
	≥ 110 g/L		44	75.5(6.6)		94.6(3.1)	
	< 110g/L		56	45.7(6.4)		71.6(5.4)	
	**Lymphocyte count**	160			0.332		0.150
	≤ 15^*^10^9/L		39	60.0(6.4)		86.4(3.9)	
	> 15^*^10^9/L		61	54.9(7.1)		73.4(6.5)	
	**Thrombocyte count**	160			0.917		0.170
	≥ 100^*^10^9/L		50	60.0(6.6)		84.0(4.8)	
	< 100^*^10^9/L		50	55.4(7.2)		78.4(5.3)	
	**Serum LDH**	160			**0.001**		0.231
	Normal		57	71.8(5.7)		84.5(4.4)	
	Elevated		43	40.3(7.5)		76.8(5.9)	
	**Serum albumin level**	152			**0.007**		**0.002**
	≥ 35 g/L		86	61.1(5.2)		84.7(3.8)	
	< 35 g/L		14	29.9(14.6)		57.4(11.6)	
	**Serum β_2_ microglobulin**	95			0.216		0.252
	≤ 3mg/L		22	82.5(11.3)		100(-)	
	> 3mg/L		78	55.9(7.3)		84.5(4.5)	
	**HBsAg**	160			**0.001**		**0.007**
	Negative		84	62.2(5.3)		88.5(3.0)	
	Positive		16	36.9(10.3)		70.8(9.4)	
	**Ann Arbor Stage**	160			0.186		0.372
	I-III		3	100(-)		100(-)	
	IV		157	56.9(4.9)		80.6(3.7)	
	**Autoimmune hemolytic anemia**	160			0.060		0.498
	Without		93	60.0(5.0)		80.4(3.8)	
	With		7	35.0(16.0)		90.9(8.7)	
	**Initial treatment**	160			**0.011**		**0.030**
	With rituximab		49	69.7(7.0)		92.1(3.4)	
	Without rituximab		51	49.1(6.3)		73.2(5.4)	
**Cytogenetic Characteristics**	**Chromosomal aberrations**	155			**0.000**		**0.005**
	Absence		75	76.0(4.4)		85.5(3.7)	
	Presence		25	38.4(9.6)		62.6(10.1)	
	**Complex karyotype**	155			**0.000**		**0.001**
	Absence		85	63.9(5.2)		86.1(3.4)	
	Presence		15	18.2(10.8)		43.4(14.1)	
	**Trisomy 12**	82			0.051		**0.000**
	Absence		89	59.2(7.8)		85.8(4.9)	
	Presence		11	40.0(17.4)		29.6(17.5)	
	**17p- (TP53)**	124			0.383		0.921
	Absence		95	58.2(5.9)		80.3(4.1)	
	Presence		5	62.5(21.3)		80.0(17.9)	
	**13q- (Rb1/ D13S25)**	122			**0.031**		0.110
	Absence		93	58.7(6.0)		82.8(4.3)	
	Presence		7	31.3(17.8)		58.3(18.6)	
	**11q23 (ATM)**	109			0.145		0.388
	Absence		97	58.4(6.3)		80.3(4.7)	
	Presence		3	0(-)		66.7(27.2)	
	**14q32 (IGH)**	126			0.440		0.620
	Absence		83	59.6(6.5)		81.0(4.6)	
	Presence		17	49.1(12.5)		79.1(9.4)	
**IGHV Genes Repertoire**	**IGHV mutation status**	39			0.313		0.726
	Unmutated		26	88.9(10.5)		90.0(9.5)	
	Mutated		74	64.2(10.1)		89.3(5.8)	
	IGHV1-2^*^04F usage	39			0.301		0.744
	Absence		77	78.8(7.7)		86.2(6.4)	
	Presence		23	38.9(20.1)		100(-)	

All cases expressed CD19 and CD20. CD5 was expressed in 26 of 151 patients (17%). Among the 160 patients, 25 patients (16%) were HBsAg-positive. Among the HBsAg-negative patients, 54 (40%) patients presented HBcAb, HBeAg or/and HBeAb positive, which indicated past exposure to HBV or occult HBV infection. Among the 54 patients, 8 patients had a documented history of chronic HBV infection, 3 patients were diagnosed as HBV-related hepatic cirrhosis, and others were occult infection. Positive anti-HCV serology is detected in 3 of 156 patients (1.9%).

### Cytogenetic aberrations

Among 160 patients, chromosome karyotypic results were not available in 5 patients due to the unanalyzable mitotic figures. Chromosomal abnormalities were detected in 39 (25%) patients, including 7 patients with single abnormalities and 24 patients with 3 or more abnormalities. The most frequently involved chromosomes were chromosome 3 (51%), 14 (26%), 18 (26%), 7 (23%). The predominant numerical aberrations were +3, -19, -18, -8, -21, +18. The most common structural changes were involved in 14q, 3p13-25, 7q, 3q25-28 and 1q.

FISH analyses identified 14q translocation in 21/126 patients (17%). Trisomy 12 was detected in 9 patients (2 concomitant with 13q deletion and 1 with 14q32 translocation). 13q14 deletion was found in 8/122 patients (6.6%). 17p13 deletion and 11q23 deletion were detected in 6 cases (5%) and 3 cases (3%), respectively.

### IGH genes repertoire and CDR3 analysis

A total of 39 SMZL patients had been successfully amplified the IGHV-IGHD-IGHJ rearrangements. Based on a cut-off value of 98% homology to the germline gene, 74% of SMZL patients were classified as mutated IGHV, with a median germline homology rate 93.40% (range from 82.35% to 97.77%). The remaining 26% of patients were identified as unmutated IGHV, of which 40% displayed 100% germline gene.

**Figure 1:**
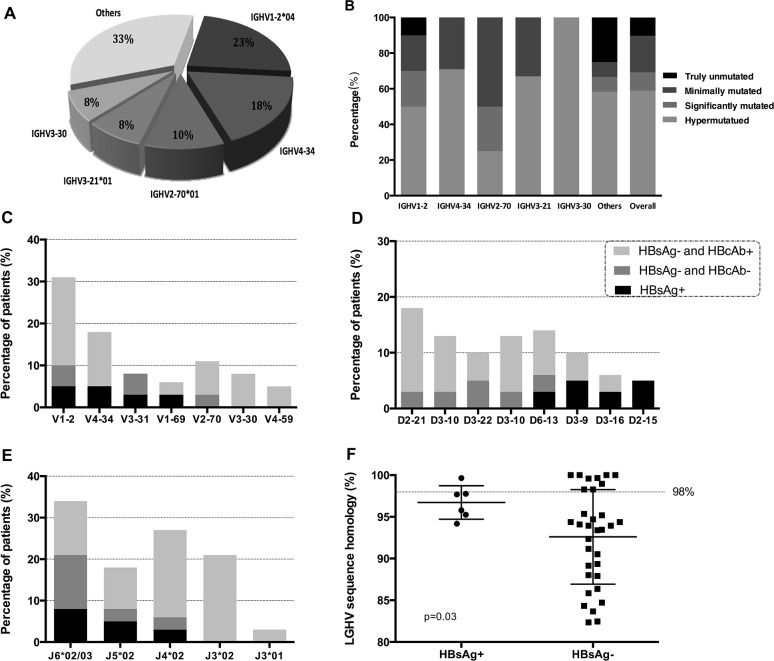
IGHV gene repertoire of the present series **(A)**. Mutational patterns of different IGHV gene subgroups **(B)**. Sequences with 100% gene identity were classified as ‘truly unmutated’ subgroup, 99.9-97% identity as ‘minimally mutated’, 96.9-95% identity as ‘significantly mutated’, <95% identity as ‘hypermutated’. IG gene repertoire and mutational status in HBsAg-positive and HBsAg-negative groups. Frequency of the IGHV subgroups **(C)**. Frequency of the IGHD subgroups **(D)**. Frequency of the IGHJ subgroups **(E)**. Mutational status in two groups **(F)**.

According to IGHV rearrangement analyses, the most frequent sequences were IGHV1-2 (26%), followed by IGHV4-34 (18%) and IGHV2-70 (10%) (Table 2). Particularly, for IGHV1-2 subgroup, gene repertoire biases were evident that 90% cases used IGHV1-2^*^04 versus the rest 10% used allele IGHV1-2^*^02. Hypermutation of IGHV were predominantly in most IGHV gene usage groups, except the IGHV2-70 group, in which minimally IGHV mutation occupied the majority (Figure 1B).

**Table 2:** list all of the IGHV\D\J usage and mutation status of 39 SMZL patients

IGHV gene	n	Unmutated/mutated	IGHD gene	n	Unmutated/mutated	IGHJ gene	n	Unmutated/mutated
V1-2	10	2/8	D2-21	7	2/5	J6^*^02	11	2/8
V4-34	7	2/5	D3-10	5	2/3	J4^*^02	10	4/6
V2-70	4	2/2	D6-13	5	3/2	J3^*^02	8	0/8
V3-21	3	0/3	D3-22	4	0/4	J5^*^02	7	4/3
V3-30	3	0/3	D3-9	4	0/4	J6^*^03	2	0/1
V1-69	2	0/2	D3-3	2	0/2	J3^*^01	1	0/1
V4-59	2	1/1	D3-16	2	0/2			
V1-3	1	1/0	D2-15	2	1/1			
V1-8	1	1/0	D1-26	2	0/2			
V3-15	1	0/1	D2-2	1	0/1			
V3-49	1	0/1	D4-23	1	0/1			
V3-66	1	0/1	D5-12	1	0/1			
V3-74	1	1/0	D5-18	1	1/0			
V4-61	1	0/1	D6-19	1	1/0			
V6-1	1	0/1						

^*^Unmutated/mutated: the ratio between identity to germline IGHV gene >98% and identity <98%

The most frequent usages of IGHD genes were IGHD3 (41%) and IGHD2 (26%). Strong biases were also evident in different IGHD alleles. IGHD 2-21^*^01/02 gene predominated (18%), followed by IGHD6-13^*^01 (13%) (Table 2). Obvious mutation status distinctions among different IGHD rearrangements were observed. IGHD6-13^*^01 gene was over-represented in the unmutated IGHV group, whereas IGHD3-9^*^01 and IGHD3-22^*^01 were more frequently in mutated IGHV group. The most frequent IGHJ subgroups were IGHJ6-02 (11 cases, 28%), followed by J4^*^02 (26%). Interestingly, all the patients with IGHJ3^*^02 rearrangement were identified as mutated IGHV group. Moreover, IGHJ4^*^02 and J5^*^02 recurred more frequently in unmutated IGHV group.

The length of IGH CDR3 ranged from 10 to 27 amino acids (median length 20). No significant differences were noted with regard to CDR3 length in different mutational status. The CDR3 length of patients with IGHV1-2^*^04 rearrangement showed significant difference from non-IGHV1-2^*^04 sequences (mean 24 vs. 19 amino acids, *P*= 0.001). Three novel subgroups with stereotyped IGH CDR3 were identified. Notably, two subgroups shared the 100% intra-subset homology, the two pattern presented usage of IGHV2-70^*^01, IGHD6-13^*^01, IGHJ5^*^02 with 98.28% germline homology, and usage of IGHV1-2^*^04, IGHD2-21^*^02, IGHJ3^*^02 with 93.94% germline homology respectively (Supplementary Table 1).

### The characteristics comparison between patients with or without HBV-Ag positive

A comparison of the clinical and laboratory characteristics of HBsAg positive and HBsAg negative patients was presented in Table 3. HBsAg positive subgroup showed significantly higher LDH lever, lower CD5 expression and less homology to the germline gene. Among the 39 patients with IGH repertoire and CDR3 analysis, 6 patients (15.4%) were HBsAg positive, and 13 patients (33.3%) were HBcAb positive. The patients with HBV infection presented biased IGHV-D-J rearrangements and mutational status (Figure 1C-1F). IGHV1 and IGHV3 were more frequently used in HBsAg positive or HBcAb positive patients. Moreover, IGHD3-9, IGHD3-16 and IGHD2-15 were more frequently in HBsAg positive group. 50% HBsAg positive patients and 71% HBcAb positive patients presented J6^*^02/03 usage. The germline homology rate of IGHV in HBsAg positive group was 97%±2.0%, which was more highly than HBsAg negative group (93%±5.7%, *P*=0.03).

**Table 3:** A comparison of the clinical and laboratory characteristics of HBsAg positive and HBsAg negative patients

Characteristics	HBsAg positive(n = 25)	HBsAg negative(n = 135)	P value
**Age (year)**	52 (2.3)	57 (1.0)	
**Gender (male/female)**	16/9	65/70	
**B symptom (with/without)**	16/9 (64%)	53/82 (39%)	
**Lymph node involvement (with/without)**	12/13 (48.0%)	61/74 (45.2%)	
**Extra nodal sites involvement (with/without)**	8/17 (32.0%)	22/113 (16.3%)	
**Hemoglobin level (g/l)**	99.6 (3.8)	107.5(2.9)	
Platelet level (^*^10^9/L)	105 (10.4)	121 (6.7)	
Lymphocyte count (^*^10^9/L)	20 (4.4)	25 (3.3)	
**ALB (g/L)**	40 (0.9)	41 (0.5)	
**LDH (U/L)**	342 (34.6)	261 (12.8)	0.016^*^
**β2 microglobulin (mg/L)**	5.6 (0.6)	4.8 (0.3)	
**Complex karyotype (with/without)**	4/20 (16.7%)	20/111 (15.3%)	
**IGHV germline homology rate**	96.7%	92.6%	0.030^*^
**ECOG (≥ 2/0-1)**	6/19 (24.0%)	25/110 (18.5%)	
**CD5 expression (positive/negative)**	0/24 (0%)	26/127 (20.5%)	0.000^**^
**IPI score (3-4 / 1-2)**	10/15(40.0%)	47/88 (34.8%)	
**Rituximab based therapy (with/without)**	8/17 (32.0%)	61/74 (45.2%)	

### Outcome and prognostic analysis

With a median follow-up of 52 months (range 6 to 214 months), 56 (35%) patients had relapsed or progressed with a median of 13 months after initial therapy (range 2 to 132 months), and 30 patients had died during the follow-up (28 with disease progression, 1 with acute heart incidents, and 1 with unknown causes). With the appropriate application of antiviral treatment, three of the patients developed HBV reactivation during lymphoma treatment (defined as increased serum HBV DNA ≥1 log10), but none of them developed a hepatitis flare. And none of the patients died of **hepatic failure or** decompensated liver cirrhosis.

**Figure 2:**
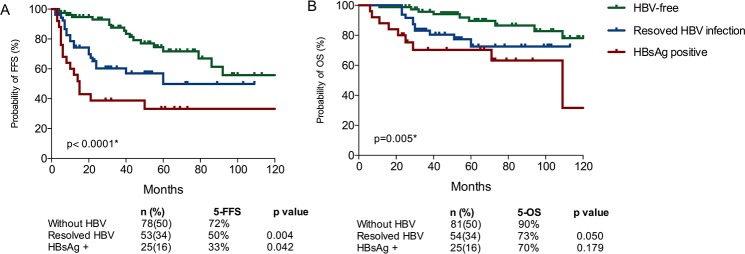
Different survival outcomes of HBsAg positive patients, resolved HBV infection patients and HBV-free patients for FFS **(A)** and OS **(B)**.

The estimated 5-year FFS and 5-year OS for the entire series were 58% (95% CI, 49%-68%) and 81% (95% CI, 75%-89%), respectively (Supplementary Figure 1). The clinical characteristics significantly associated with shorter survival include ECOG ≥ 2, Hemoglobin < 110g/L, albumin level < 35 g/L, HBsAg-positive, initial treatment without rituximab and without maintenance therapy (Table 1). HBsAg-positive patients had poorer outcome than HBsAg-negative ones. In fact, patients with resolved HBV infection (HBsAg negative and hepatitis B core antibody [anti-HBc]-positive) had a worse survival than patients who were free from HBV infection (all items negative or only hepatitis B surface antibody [anti-HBs]-positive) (5-year FFS, 49.8% vs. 71.7%, p=0.004; 5-year OS, 72.6% vs. 89.6%, p=0.050) (Figure 2). Notably, the tradition adverse prognostic factors such as age, LDH or CD5 expression were not identified in this analysis.

**Figure 3:**
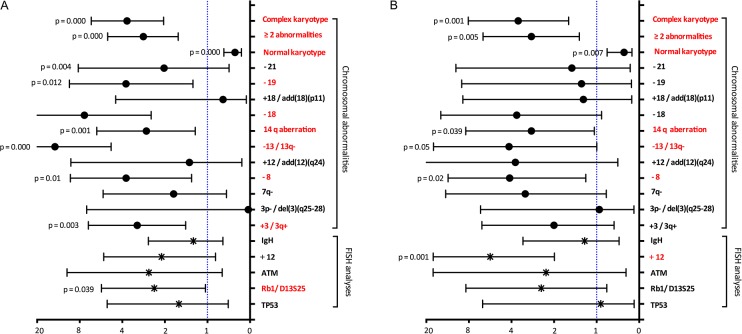
Hazard ratios obtained by univariate Cox analysis for FFS **(A)** and OS **(B)** according to recurrent chromosomal abnormalities and FISH analyse.

Patients with chromosome karyotypic aberrations showed a poor survival and patients with complex karyotype had a poorer survival (Table 1). For the recurrent abnormalities, a significantly shorter survival was noted for the presence of structural abnormalities at 14q aberration (HR=2.5, 95%CI 1.1-4.8, P=0.039). -13/13q- (HR=3.0, P=0.02) and -8 (HR=3.1, P =0.05) were also significant association with shorter survival. Besides, patients with +3 / 3q+, -18, and -19 showed a significant correction of shorter FFS (Figure 3). Based on FISH analyses, patients with trisomy 12 had a shorter survival, whereas, no significant associations with survival were identified for del13q, del11q deletion and IGH translocation. Moreover patients with del 13q had a higher risk of progression. Unexpectedly, IGHV mutation status showed no prognostic impact on both FFS (p=0.31) and OS (p=0.73). Also, survivals were similar among different IGHV usage subgroups (Table 1).

**Figure 4:**
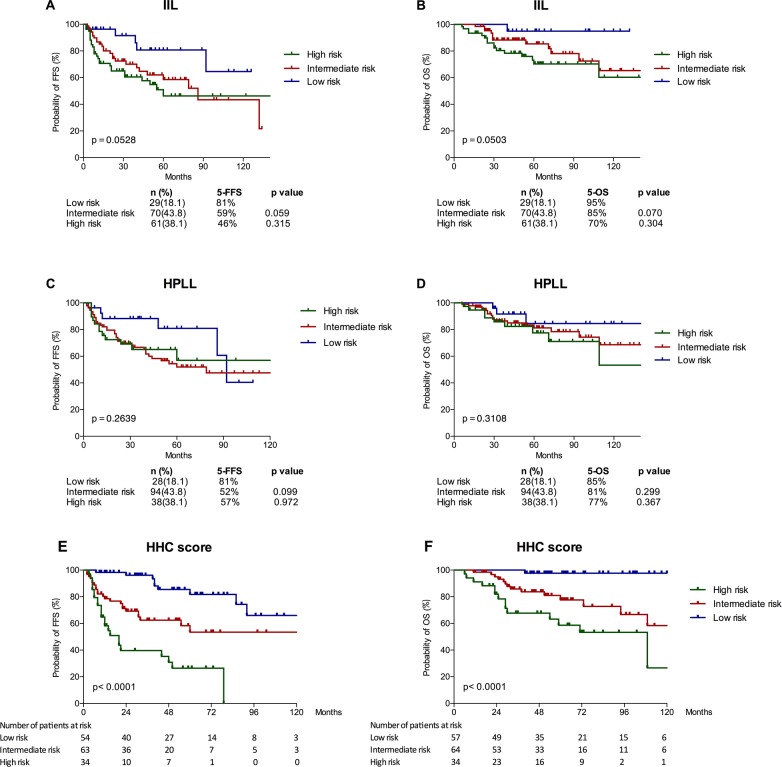
Survival outcomes of patients in different risk groups defined based on the Intergruppo Italiano Linfomi (IIL) score **(A, B)**, the model proposed by SMZLSG based on Hb, platelet count, LDH and lymphadenopathy (HPLL score) **(C, D)** and the new prognostic score (HHC score) **(E, F)**.

Based on the results from the multivariate analysis, a three-factor prognostic model (Hb < 110g/l, HBsAg positive, and complex karyotype, named “HHC score”) was constructed with one point assigned to each factor (Supplementary Table 2). There were only two patients with 3 score. Thus, patients with a score of 2-3 were classified as high-risk (n=34, 22%), score of 1 as intermediate-risk (n=64, 41%), and score of 0 as low-risk (n=57, 37%). This prognostic model can distinguish these patients into three risk groups with 5-year estimated OS rates of 98% (low risk), 78% (intermediate risk), and 59% (high risk) (P<0.001) and with 5-year estimated FFS of 82%, 53% and 26%, respectively (P<0.001) (Figure 4E, 4F).

We then evaluated the prognostic role of the IIL and HPLL score systems, which was established on Caucasia population. The two models failed to discriminate our cohort (Figure 4A-4D). Conversely, significant OS and PFS differences were found in all the risk groups using the novel HHC score (Table 4). C-index of the HHC model for OS is significant better than IIL model (0.750 vs. 0.669, p=0.045) and HPLL model (0.750 vs. 0.597, p=0.000). The novel SMZL model was also the most accurate prognostic system for FFS based on the highest C-index, followed by the IIL and HPLL models (0.736, 0.640 and 0.567 respectively) (Supplementary Table 3).

**Table 4:** OS and FFS of patients in different risk groups defined based on IIL and HPLL scores and the new three-factor HHC model

Models (score)	Patient, n (%)	5-Year FFS	P value	5-Year OS	P value
**IIL score**			0.053		0.050
Low risk (0)	29 (18%)	81%		95%	
Intermediate risk (1)	70 (44%)	59%	0.059	85%	0.070
High risk (2-3)	61 (38%)	46%	0.315	70%	0.304
**HPLL score**			0.264		0.311
Low risk (0)	28 (18%)	81%		85%	
Intermediate risk (1-2)	94 (44%)	52%	0.099	81%	0.299
High risk (3-4)	38 (38%)	57%	0.972	77%	0.367
**HHC score**			P<0.001^**^		P<0.001^**^
Low risk (0)	57 (37%)	82%		98%	
Intermediate risk (1)	64 (41%)	53%	0.001^**^	78%	0.000^**^
High risk (2-3)	34 (22%)	26%	0.003^**^	59%	0.032^*^

^*^Significant P value (< 0.05) for two neighboring risk groups, ^**^ p value <0.01

## DISCUSSION

In this study, we described the clinical and biological characteristics of SMZL in HBV prevalent region and developed a novel hierarchical prognostic model for this population. According to our knowledge, this is the first report to investigate SMZL derived from Eastern population with large series patient.

Compared with Western reports [6–11], younger age, more common presence of B symptom, higher LDH level and lower thrombocyte counts at diagnosis were observed in our population. Notably, the positive rate of HBsAg was 16%, which is significantly higher than 7.1% in the normal population infection level in China [12]. However, a higher incidence of HCV infection (8%-19%) has been reported in patients of European countries [2, 3, 6]. Growing evidence indicates that HCV is closely correlated with the pathologic mechanisms of SMLZ. The E2 glycoprotein of the HCV could interact with CD81 in B cells, thereby leading to a proliferation of B cells [13]. Literature surrounding an association with Hepatitis B virus (HBV) and SMZL is minimal with a few case reports documenting a possible association [14, 15]. In our study, we found HBsAg-positive SMZL patients showed unique clinical features, including higher LDH level, more common B symptom, much less CD5 expression rate, less homology to the germline gene and poorer outcome. These could provide evidence that HBsAg-positive SMZL is a distinct population, and chronic HBV-associated antigen stimulation might contribute to the pathogenesis of SMZL. The hypothesis was substantiated by the different usage of IG genes among different HBV infection status (Figure 1). However, apparent differences should be demonstrated between HCV and HBV-associated SMZL. The direct relationship between HCV infection and SMZL is strongly supported by the neoplasm regression following antiviral therapy. But no lymphoma response to antivirus therapy was found in HBV-associated SMZL. Moreover, the difference between RNA virus and DNA virus might result in different biologic features and clinical responses. The pathologic role that HBV plays in the development of SMZL is currently unclear. Chronic HBV infection is characterized by the presence of abundant viral proteins, and antibody responses are vigorous and sustained. HBV antigen-driven chronic inflammatory process that leads B cells from polyclonal to monoclonal expansion might be an initiating event in the lymphomagenesis of HBV-infected SMZL. The exact mechanisms need to be further explored.

Then, we performed an immunogenetic analysis of IGHV-IGHD-IGHJ rearrangements to determine whether there was a biased IG gene repertoire responding to the involvement of antigens in lymphomagenesis. The preferential use of IGHV1-2^*^04 and IGHV4-34 was found in our data correspond to the report of other series [16, 17]. However, frequent use of IGHV2-70^*^01 and IGHV3-21^*^01 were never reported before and IGHV3-23 usage which was employed over-representation (8%-25%) in North European was not found in our series [16–19]. Similarly, IGHD segment spectrums also showed distinction with more preferential use of IGHD2-21 and sporadic use of IGHD3-3 [16–18]. This might attribute to the different immunology background and infectious spectrum in China. In addition to the biased usage and mutational status of IGHV, three SMZL-specific stereotyped CDR3 sequences were first reported in our study. Some of the cases shared the totally identical IGH CDR3 sequences, suggesting a potential typical pathogenic trigger in different patients. But the interesting thing is that these stereotyped CDR3 sequences all occurred in HBsAg-negative cases. Thus, we could speculate that the hepatitis infection spectrum may partly explain the different IgHV repertoire in china, but not the whole story. There might be some HBV-unrelated epitopes stimulating proliferation of B cells and express the particular IGH genes. It may offer promising research directions for further study.

Our data also showed that patients with SMZL had variable cytogenetic aberration. A lower proportion of cases with del17p and del13q were detected by FISH (5% and 7% respectively) compared to the proportion of 18% and 40% reported in the study of European population [3]. IGH translocations detected by FISH were 21/126 (17%), more common than other population (5/61, 8%) [3]. The different genetic behavior might account for the distinct clinical and laboratorial characteristics in Chinese patients with SMZL. Complexity of the karyotype was used to propose as poor prognostic indicators but failed to retained prognostic significance for OS on multivariate analysis in Western report [3]. In our study, complex karyotype is an independent prognostic factor for survival. Notably, our findings did not copy the results from previous studies, which reported an unfavorable predictor for del17p [3, 20, 21] or 7q deletion [22]. Instead, we provided evidence to suggest that trisomy 12, 14q aberration and 13q deletion have adverse impacts on survival. Similarly, the loss of the TP53 or 17p was reported no prognostic impact of SMZL in several studies [23, 24]. Larger series were needed to further validate the true prognostic impact of karyotype abnormality. Furthermore, there is no consensus on the prognostic impact of IGHV mutations. Some studies have confirmed a more aggressive clinical course in patients with unmutated IGHV genes [20, 22], while other studies reported contrary findings [16, 25].

Several prognostic factors have been identified for SMZL by different investigators (Table 5). Most of previous studies were developed for European patients, and took splenectomy as their dominating therapy options. This present study is the first one to propose prognostic factor in Chinese patients. Moreover, almost half of our series chose rituximab-based immunochemotherapy as first-line therapy. We failed to find prognostic significance for traditional risk factors, such as age, lymphocytosis, β2-microglobulin or LDH, which can be attributable to the distinct clinical and cytogenetic spectrums in our series or maybe associated with the much higher proportion of rituximab application. The independently inferior survival impact of HBV infection is first identified in our study. Similar conclusion was reached in the series of 309 Italy patients [2]. They found patients with HBV infection had a higher risk of progression, but HBsAg was positive in only 13 (5%) patients and no independent prognostic significance was found in that study [2]. Even in the era of rituximab, the treatment options did not influenced by HBV infection. Few patient had fulminant hepatitis during immunochemotherapy with programmed preventative anti-virus drugs administration. And few of them had adverse outcomes due to hepatic failure or liver-related complications. Thus, the adverse prognosis of HBV infection patients could not be explained by rituximab-treatment limitation or liver toxicity. Moreover, besides active chronic HBV infection, patients in our study with resolved HBV infection also showed inferior outcomes than HBV-free patients. The different clinical course of the three HBV infection status groups may suggest that HBV infection was the unique prognostic factor of SMZL.

**Table 5:** Prognostic factors for SMZL in different series

Study	Country	n	Prognostic factors for OS or CSS	
			In univariate analysis	In multivariate analysis
Chacón JI, et al, 2002 [31]	Spain	60	Response to therapy, nonhematopoietic sites involvement, p53 expression, ECOG≥2	Response to therapy, nonhematopoietic sites involvement
Thieblemont C, et al, 2002 [32]	France	81	M component, β2-microglobulin, lymphocytosis	-
Lenglet J, et al, 2014 [33]	France	100	Histological transformation, age	-
Parry-Jones N, et al, 2003 [34]	UK	129	Hb, lymphocytosis, PLT and hepatomegaly	Hb, lymphocytosis
Xing KH, et al, 2015 [7]	England	107	Hb, LDH, age, ECOG≥2	LDH, age
Perrone S, et al, 2016 [11]	Italy	100	Female-sex, splenomegaly, ECOG≥1	ECOG≥1
Arcaini L, et al, 2006 [2]	Italy	309	Hb, ALB, LDH, age, PLT and HbsAg-positivity	Hb, LDH, ALB *(IIL prognostic model)*
Montalban C, et al, 2012 [6]	Spain, Italy and UK	593	Hb, PLT, LDH, lymphadenopathy, Age	Hb, PLT, LDH and lymphadenopathy *(HPLL prognostic model)*
**Our series**	China	160	Hb, ECOG ≥ 2, ALB, HbsAg-positivity, complex karyotype, initial treatment without rituximab and without maintenance therapy	Hb, HbsAg-positivity, complex karyotype *(HHC prognostic model)*

Then, we tested the established prognostic soring systems in this population. The two well-known scoring systems, IIL and HPLL systems, did not perform the expectant effect to distinguish hierarchical survival in our patients. We identified three risk factors as independently prognostic factors and provided a new stratification named HHC scores of three groups with different outcomes. Hemoglobin level was considered in both IIL score [2] and HPLL score [6]. But HBsAg-positivity and complex karyotype have not yet been considered in previous prognostic models of SMZL. Among the three prognostic models, only the c-index of our HHC score was over 0.7 in our series (Supplementary Table 3). As to the IIL and HPLL score, which were developed on the basis of cohorts in European countries with high HCV prevalence, the c-index of the two scores were relative lower, and there were no significant differences in OS were found among the three risk groups. Hence the previous models are not adequate to establish prognosis in our series. Moreover, for the high-risk group according to HHC score, patients received splenectomy (n=13) had a more progressive course than patients received rituximab-based immuochemotherapy (n=11) (p=0.049, 5y-FFS: 9.1% vs. 51.9%; 5y-OS: 53.0% vs. 80.8%, p=0.131). However, whether it could be used to develop risk-tailored treatment approaches for patients with SMZL should be further examined, and the prognostic significance of this model should be verified by future prospective studies.

Our study had several limitations. First, our data were collected from single center in the Eastern population with HBV prevalent region. Additionally, the absence of a validation cohort limited the application value of the HHC score. Thus, we advise extreme caution to apply the prognostic model in other population, especially in HCV epidemic population. Second, IGH rearrangement analyses were performed in only 39 SMZL cases. The distinctive IGH usage and CDR3 sequences of HBV-infective SMZL need to be checked up in further study with enlarged sample size.

Our data demonstrated the distinctive clinical characteristics, viral background, IGHV repertoire and cytogenetic aberrations in Chinese patients with SMZL. Patients with low hemoglobin level, HBV infection and complex karyotype had much inferior survival. A new HHC prognostic scoring system was proved as the most informative predictor in our series. It may also be helpful in selecting the appropriate treatment strategies for individual patients.

## MATERIALS AND METHODS

### Patients

In total, 160 patients were diagnosed with SMZL between September 2001 and September 2015 in the Institute of Hematology and Blood Disease Hospital, Chinese Academy of Medical Sciences and Peking Union Medical College. Diagnosis was established according to 2008 WHO classification criteria [1]. In patients with clinical splenomegaly and splenectomy is not available, the diagnosis was established according to SBLG guidelines [26], based on a combination of clinical presentation, lymphocyte morphology, immunophenotype, and typical intrasinusoidal infiltration in bone marrow (BM) histology. The diagnoses were reviewed by two hematopathologists (E.L. and K.L.). Clinical database on age, sex, clinical symptom, involving sites, laboratory findings, virus status, bone marrow histology, immunohistochemistry, immunophenotype by flow cytometry (FCM), recurrent chromosomal abnormalities before first-line treatment were retrospectively analyzed.

### Cytogenetic analysis and IGH genes usage

Chromosome karyotype analyses were performed on bone marrow cells, cultured for 24h at 37°C without stimulation. G-banded technique was used. Interphase fluorescence in situ hybridization (FISH) analysis was performed on peripheral blood or bone marrow samples at diagnosis. The DNA probes included the loci centromere 12 (CEP12), 13q14.3 (LSI D13S25 and RB-1), 14q32 (LSI IGHC/IGHV), 17p13 (LSI TP53), and 11q22 (LSI ATM). Sample preparations and hybridizations were conducted following the manufacturer's recommendations and as previously described [27, 28]. IGH genes usage were detected Sanger sequencing as previous described [29].

### Treatment

For each patient, all lines of treatment were recorded. Five patients were observed without any treatment until the follow-up endpoint. Splenectomy was performed in 45 patients (followed by no treatment, 22; chemotherapy, 13; rituximab-based immunochemotherapy, 10). 30 patients received chemotherapy only (cyclophosphamide, doxorubicin, vincristine, and prednisone [CHOP], 22; etoposide + CHOP [CHOEP], 3; fludarabine-based regimen, 3; others, 2). 69 received rituximab-based immunochemotherapy (rituximab with CHOP regimen, 44; COP regimen, 14; fludarabine-based regimen, 7; CHOEP regimen, 3; Ibrutinib, 1). 11 patients received alkylating agent only. 79 patients with active or resolved HBV reaction and three with HCV infection were treated with preventive antiviral therapy (entecavir, lamivudine or interferon-a), which was not considered to be definitive therapy when calculating FFS.

### Statistical analysis

Overall survival (OS) was calculated from time of diagnosis to time of death from any cause or to last follow-up. Failure- free survival (FFS) was measured from the date of first therapy initiation to the date of first relapse/progression, transformation, death or last follow-up. Comparison of clinical characteristics between patients with normal karyotypes and with complex karyotypes, or between HBsAg positive and HBsAg negative patients was evaluated using student t-test, χ2 test or Fisher's exact test. Survival curves were constructed using the Kaplan-Meier method. The effects of potential prognostic variables on survival were assessed according to the Cox regression method.

We then selected risk factors in our prognostic score system by applying the forward stepwise method with a selection criterion of p value less than 0.05. Because of the comparable hazard risks of the three selected factors, we identified the same weight of all factors in our score. The prognostic value and discriminatory ability of the score were assessed by the index of concordance (c-index). A higher c-index indicates a more explanatory and informative model. We made a comparison with the previous prognostic system of SMZL included IIL score and HPLL score using R-studio 0.99.903 and R 3.3.1 by the method of Frank Harrell [30]. *P* values <.05 were considered statistically significant. All calculations were performed using the SPSS statistical software package (Version 21.0, Inc., Chicago, IL).

## SUPPLEMENTARY MATERIALS FIGURE AND TABLES



## Abbreviations

SMZL: splenic marginal zone lymphoma
FISH: fluorescence in situ hybridization
OS: overall survival
CCS: cause-specific survival
FFS: Failure- free survival
IGHV: immunoglobulin heavy variable
IIL: Intergruppo Italiano Linfomi
HCV: hepatitis C virus
HBV: hepatitis B virus
HBsAg: hepatitis B surface antigen
Hb: hemoglobin
LDH: lactate dehydrogenase
ALB: albumin
PLT: Platelet count;
