# Supplementary material for: Distinct clinical characteristics draw a new prognostic model for splenic marginal zone lymphoma in HBV high prevalent region
Source: Oncotarget. 2017 Oct 19;8(58):98757–70. doi: 10.18632/oncotarget.21931 (PMC5716765; doi:10.18632/oncotarget.21931)
Supplement: Supplementary file 1 [file oncotarget-08-98757-s001.pdf]

Distinct clinical characteristics draw a new prognostic model for splenic marginal zone lymphoma in HBV high prevalent region

SUPPLEMENTARY MATERIALS

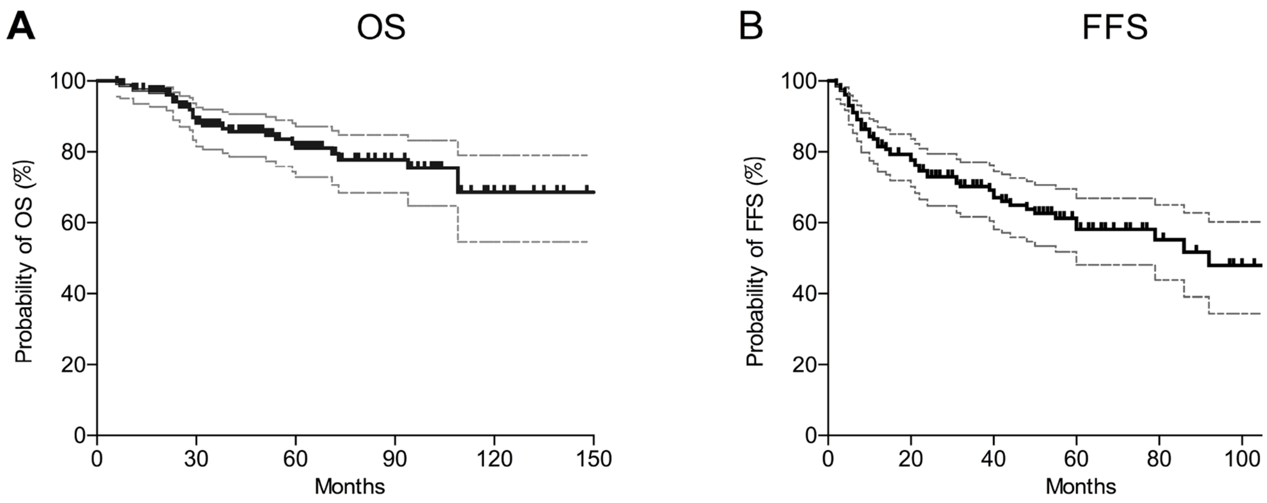

Supplementary Figure 1: OS **(A)** and FFS **(B)** curve for the whole series, the double dash lines accounted for 95% confidence interval.

Supplementary Table 1: Subsets of SMZL cases with stereotyped CDR3 sequences

Subset	Average intra-subset identity	IGHV	IGHD	IJHK	Germline IGHV gene identity	CDR3 amino acids sequence	Amino acids lenth
1	100%	IGHV2-70*01	IGHD6-13*01	IGHJ5*02	98.28%	CARTYSIVAVGTPAWFDPW	19
		IGHV2-70*01	IGHD6-13*01	IGHJ5*02	98.28%	CARTYSIVAVGTPAWFDPW	19
2	100%	IGHV1-2*04	IGHD2-21*02	IGHJ3*02	93.94%	CARGTENIVVVTATRSGPHDAFDIW	25
		IGHV1-2*04	IGHD2-21*02	IGHJ3*02	93.94%	CARGTENIVVVTATRSGPHDAFDIW	25
3	65%	IGHV1-8*01	IGHD6-13*01	IGHJ4*02	100%	CARSAAAGTVWSLHYGMDVW	20
		IGHV1-2*04	IGHD6-19*01	IGHJ6*02	100%	CAKGIAVAGTQYYYYGMDVW	20

Supplementary Table 2: Clinical parameters influencing OS and FFS in Cox's regression analysis

	FFS *		OS †	
	HR (95%CI)	P value	HR (95%CI)	P value
**Hemoglobin level < 110g/L**	2.71(1.39-5.29)	0.003	5.86(1.76-19.55)	0.004
**HBV infection**	2.97(1.62-5.46)	0.000	2.81(1.25-6.31)	0.012
**Complex karyotype**	2.69(1.43-5.07)	0.002	3.03(1.34-6.89)	0.008

HR, hazard risk; CI, confidence interval.
* Model χ^2 = 39.510, P<0.001, † Model χ^2 = 28.660, P<0.001

Supplementary Table 3: Comparison of the prognostic abilities of the three prognostic models with C-index

Prognostic model	FFS			OS		
	C-index	95%CI	P value	C-index	95%CI	P value
**HHC score**	0.736	0.696-0.776		0.750	0.695-0.805	
**IIL score**	0.640	0.599-0.681	0.160	0.669	0.613-0.725	0.045
**HPLL score**	0.567	0.525-0.609	0.000	0.597	0.539-0.655	0.000
